# Dynamic Membrane Localization of RNase Y in Bacillus subtilis

**DOI:** 10.1128/mBio.03337-19

**Published:** 2020-02-18

**Authors:** Lina Hamouche, Cyrille Billaudeau, Anna Rocca, Arnaud Chastanet, Saravuth Ngo, Soumaya Laalami, Harald Putzer

**Affiliations:** a UMR 8261, CNRS, Université de Paris, Institut de Biologie Physico-Chimique, Paris, France; b Micalis Institute, INRA, AgroParisTech, Université Paris-Saclay, Jouy-en-Josas, France; Institut Pasteur

**Keywords:** *Bacillus subtilis*, RNA degradation, RNA processing, RNase Y, membrane proteins

## Abstract

Metabolic turnover of mRNA is fundamental to the control of gene expression in all organisms, notably in fast-adapting prokaryotes. In many bacteria, RNase Y initiates global mRNA decay via an endonucleolytic cleavage, as shown in the Gram-positive model organism Bacillus subtilis. This enzyme is tethered to the inner cell membrane, a pseudocompartmentalization coherent with its task of initiating mRNA cleavage/maturation of mRNAs that are translated at the cell periphery. Here, we used total internal reflection fluorescence microscopy (TIRFm) and single-particle tracking (SPT) to visualize RNase Y and analyze its distribution and dynamics in living cells. We find that RNase Y diffuses rapidly at the membrane in the form of dynamic short-lived foci. Unlike RNase E, the major decay-initiating RNase in Escherichia coli, the formation of foci is not dependent on the presence of RNA substrates. On the contrary, RNase Y foci become more abundant and increase in size following transcription arrest, suggesting that they do not constitute the most active form of the nuclease. The Y-complex of three proteins (YaaT, YlbF, and YmcA) has previously been shown to play an important role for RNase Y activity *in vivo*. We demonstrate that Y-complex mutations have an effect similar to but much stronger than that of depletion of RNA in increasing the number and size of RNase Y foci at the membrane. Our data suggest that the Y-complex shifts the assembly status of RNase Y toward fewer and smaller complexes, thereby increasing cleavage efficiency of complex substrates like polycistronic mRNAs.

## Abstract (precis)

All living organisms must degrade mRNA to adapt gene expression to changing environments. In bacteria, initiation of mRNA decay generally occurs through an endonucleolytic cleavage. In the Gram-positive model organism Bacillus subtilis and probably many other bacteria, the key enzyme for this task is RNase Y, which is anchored at the inner cell membrane. While this pseudocompartmentalization appears coherent with translation occurring primarily at the cell periphery, our knowledge on the distribution and dynamics of RNase Y in living cells is very scarce. Here, we show that RNase Y moves rapidly along the membrane in the form of dynamic short-lived foci. These foci become more abundant and increase in size following transcription arrest, suggesting that they do not constitute the most active form of the nuclease. This contrasts with RNase E, the major decay-initiating RNase in E. coli, where it was shown that formation of foci is dependent on the presence of RNA substrates. We also show that a protein complex (Y-complex) known to influence the specificity of RNase Y activity *in vivo* is capable of shifting the assembly status of RNase Y toward fewer and smaller complexes. This highlights fundamental differences between RNase E- and RNase Y-based degradation machineries.

## INTRODUCTION

Degradation and processing of mRNA are important steps in the control of gene expression, notably in fast-growing bacteria (1–4). The decay of a bacterial mRNA generally follows an all-or-none pattern, implying that if control is to be efficient, it must occur at the initiating step. Studies in the two model organisms, Escherichia coli and Bacillus subtilis, distant by 3 billion years of evolution (5), have been instrumental for the discovery of the major enzymes involved in this process (6). Almost all known bacteria have RNase E and/or RNase Y, which are completely different proteins but functionally similar endoribonucleases that emerged by convergent evolution (2). In addition, these proteins have distinctive subcellular addresses, with RNase E forming cytoplasmic P-body-like assemblies in *Caulobacter* (7, 8) or short-lived membrane-tethered foci in E. coli (9, 10). In B. subtilis, RNase Y is also bound to the membrane, probably by a single-pass N-terminal helix (11; RNase Y is called YmdA in this reference), where it might also interact with the dynamin-like protein DynA (12). This subcellular localization fits well with the observation that translation occurs predominantly on ribosomes distributed around the cell periphery (13, 14). It highlights the capacity of bacteria to establish an intricate subcellular architecture in which important biological processes can be confined to microenvironments (15, 16).

B. subtilis RNase Y affects global mRNA stability; the protein shows endonucleolytic activity on preferably 5′ monophophorylated substrates *in vitro* with an RNase E-like single-strand-specific cleavage specificity (17). It affects the intracellular levels of a majority of transcripts in B. subtilis (18–20) and S. pyogenes (21) but does so to a lesser extent in S. aureus (9). The existence of an RNase Y-based degradosome has been proposed (22, 23), but in contrast to the E. coli (24, 25) and Caulobacter crescentus (26) RNase E-based degradosomes, such a complex cannot be isolated from B. subtilis in the absence of cross-linking agents. Whether RNase Y can form any meaningful interactions with other ribonucleases *in vivo* remains an open question (2, 27–29).

Recently, three small proteins, YlbF, YmcA, and YaaT, were shown to alter RNase Y activity *in vivo* (30). These proteins can stably bind to each other, forming the so-called Y-complex (31, 32), and are required for the efficient maturation of operon mRNAs and affect the abundance of certain riboswitches (33). They were shown to be important for sporulation, natural competence for transformation, and biofilm formation (34–38). The Y-complex also carries two oxygen-sensitive [4Fe-4S]^2+^ clusters (39) that are important for their pleiotropic functions (32). To what extent these diverse effects of the Y-complex are specific or indirect, by modulating the RNase Y-mediated mRNA stability, is unknown. RNase Y was one of the proteins found in independent pulldown experiments using YlbF (30) and YaaT (31). Since it does not affect all RNase Y targets, the Y-complex has been proposed to act as a specificity factor for this general endonuclease (33).

To examine how the Y-complex accomplishes this feat, we first studied the distribution and dynamics of the membrane-bound RNase Y using time-lapse total internal reflection fluorescence microscopy (TIRFm), along with single-particle tracking (SPT) analysis. RNase Y was found to form discrete short-lived foci, assemblies, that diffuse freely along the cell periphery, showing different dynamic behaviors. Without the N-terminal transmembrane domain, RNase Y is uniformly distributed in the cytoplasm. However, while a similar transient clustering of RNase E into cooperative active degradation bodies was observed in E. coli (10), RNase Y focus formation is, in contrast to that of RNase E, independent of the presence or the abundance of RNA substrates. Rather, our observations suggest that clustering of RNase Y represents an inactive or less active form of the enzyme that accumulates in the absence of substrate. In agreement, we show that the three Y-complex proteins are required to maintain the number of RNase Y foci at a lower level and to reduce their size. This work suggests that the Y-complex modulates the specificity of RNase Y by altering the membrane assembly status of the endoribonuclease.

## RESULTS

### RNase Y localization at the membrane is highly dynamic.

**FIG 1:**
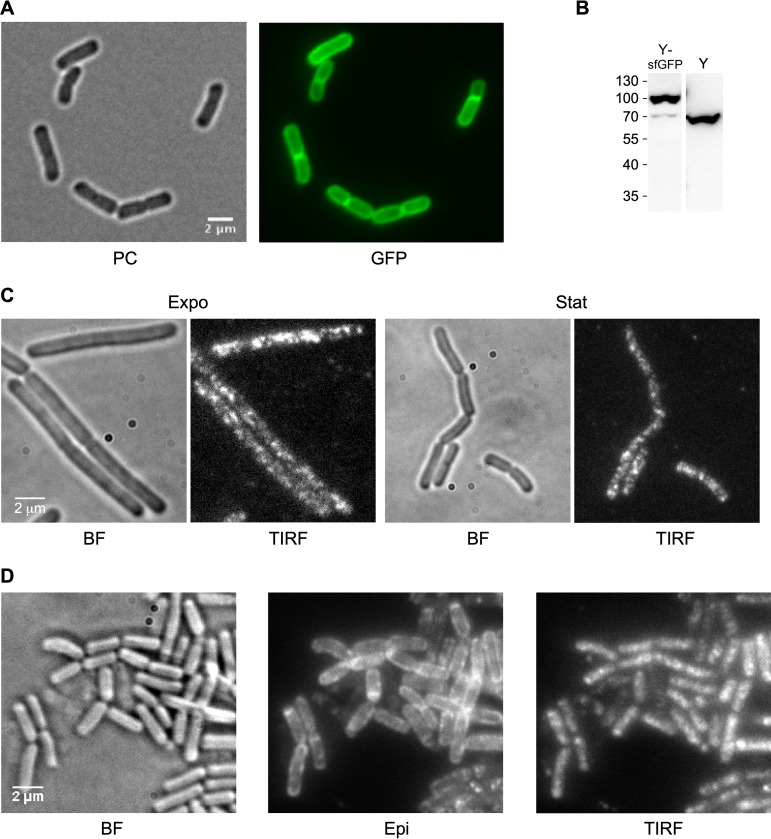
Visualization of RNase Y in the cell. (A) RNase Y localizes at the membrane. Strain SSB2063a (P*_xyl_-rny-mgfpmut1*) was grown in SMS minimal medium at 37°C to early stationary phase in the presence of 60 mM xylose to induce expression of the RNase Y-GFP fusion protein and observed by phase-contrast (PC) and wide-field fluorescence microscopy (GFP). (B) Quantification of RNase Y-sfGFP and RNase Y expression by Western blotting. Total cell extracts of strains SSB2048 and SSB1002 (WT) were analyzed using an anti-RNase Y monoclonal antibody. For further details, see the legend to Fig. 6D. (C) RNase Y focus formation at the membrane. Cells of strain SSB2063a grown in LB medium at 37°C and expressing RNase Y-GFP fusion protein were imaged at mid-log (Expo) and stationary phase (Stat). The focus pattern of RNase Y-GFP fusion protein was visualized by TIRF microscopy (100-ms exposure time). BF, bright field. (D) RNase Y-sfGFP (construct at the *rny* locus, SSB2048) dynamically localizes in membrane foci. The cultures were grown to mid-log phase in the SMS minimal medium at 37°C. The TIRF images were extracted from 30-s time-lapse movies. All images were acquired on living cells deposited on an agar pad and covered by a glass slide. Scale bar, 2 μm. BF, bright field; Epi, epifluorescence; TIRF, total internal reflection fluorescence.

RNase Y has a single transmembrane domain at the N terminus, which anchors the protein at the membrane (11) with the C-terminal part of the protein located in the cytosol. In order to localize RNase Y in living cells, we ectopically expressed a C-terminal RNase Y-green fluorescent protein (GFP) fusion protein from a xylose-inducible construct integrated at the *amyE* locus. This strain (SSB2063a) has no discernible phenotype under the experimental conditions used. As expected, epifluorescence microscopy showed that the bulk of RNase Y was localized at the cell membrane (Fig. 1A).

We then analyzed the same strain using TIRF microscopy. This technique produces images with improved signal-to-noise ratios of peripherally localized proteins by selectively exciting fluorophores close to the glass-specimen interface. It is, therefore, a sensitive technique for studying the dynamics of molecules near the cell surface, like RNase Y, with high temporal resolution. SSB2063a cells, grown in LB at 37°C in the presence of xylose to induce *rny-gfp* expression, were analyzed at early exponential and stationary phase (Fig. 1C). We observed that RNase Y is not uniformly distributed along the membrane, as suggested by epifluorescence images (Fig. 1A). Instead, RNase Y formed distinct foci (assemblies) along the rod in the form of asymmetrical, variably sized foci (Fig. 1C). They appeared within the diffraction limit that restricts the lateral resolution to 200 to 300 nm and the axial resolution to 100 to 200 nm (40). These foci circulated in a fluctuating and rapid movement at the cell periphery (see Movie S1 in the supplemental material). It has been shown previously that fluorescent proteins can aggregate, which can affect the subcellular localization pattern, but the presence of the monomeric A206K mutation in GFP inhibits or strongly reduces this effect (41). We have compared two strains expressing RNase Y-GFP fusions based on the GFPmut1 protein. One strain (SSB2063b) carried the unmodified GFPmut1 open reading frame (ORF), while the other (strain SSB2063a) expressed GFPmut1 carrying the A206K mutation (mGFPmut1). Both strains showed an identical or very similar membrane localization of RNase Y with the formation of well distinguishable foci (Fig. S1A and compare Movies S1 and S2).

10.1128/mBio.03337-19.1 FIG S1 RNase Y only forms foci when tethered to the membrane. (A) Cells of strain SSB2063a, expressing RNase Y-mGFPmut1 (left), and SSB2063b, expressing RNase Y-GFPmut1 (right), were grown in SMS medium at 37°C and imaged at mid-log phase by TIRF microscopy (100-ms exposure time). (B) Strain SSB2066 expressing the ΔTMD-RNase Y-sfGFP fusion protein grown in LB medium at 37°C and imaged by epifluorescence (Epi) and bright-field microscopy (BF). Scale bar, 2 μm. Download FIG S1, PDF file, 1.2 MB. Copyright © 2020 Hamouche et al. 2020 Hamouche et al. This content is distributed under the terms of the Creative Commons Attribution 4.0 International license.

10.1128/mBio.03337-19.7 MOVIE S1 TIRF time lapse acquisition of RNase Y-mGFPmut1 A206K in live cells (strain SSB2063a, mid-log phase in SMS minimal medium at 37°C) in continuous illumination mode (duration, 30 s; exposure time, 100 ms). RNase Y-mGFPmut1 (A206K) forms very dynamic fast-moving foci at the cell periphery. Download Movie S1, AVI file, 0.5 MB. Copyright © 2020 Hamouche et al. 2020 Hamouche et al. This content is distributed under the terms of the Creative Commons Attribution 4.0 International license.

10.1128/mBio.03337-19.8 MOVIE S2 TIRF time lapse acquisition of RNase Y-GFPmut1 in live cells (strain SSB2063b, mid-log phase in SMS minimal medium at 37°C) in continuous illumination mode (duration, 30 s; exposure time, 100 ms). RNase Y-GFPmut1 forms very dynamic fast-moving foci at the cell periphery. Download Movie S2, AVI file, 0.6 MB. Copyright © 2020 Hamouche et al. 2020 Hamouche et al. This content is distributed under the terms of the Creative Commons Attribution 4.0 International license.

The distribution of these assemblies seems very similar in both exponential- and stationary-phase cells, but foci were slightly bigger and brighter in the latter (Fig. 1C). This apparent accumulation of RNase Y during stationary phase could be an artifact resulting from its expression from an inducible promoter, as recently shown with the MreB-Gfp fusion in B. subtilis (42). In order to rule out such an effect, we constructed a strain (SSB2048) expressing an RNase Y-superfolder GFP (sfGFP) fusion protein from the original chromosome location using the native *rny* promoter. We used the superfolder version of GFP, which folds more efficiently even when fused to a poorly folding protein (43, 44). Strain SSB2048, expressing RNase Y-sfGFP as the sole source of RNase Y, appeared functional: it displays a wild-type (WT) morphology in exponential and stationary phases of growth (Fig. S2B) and has only a slightly increased doubling time compared to that of a wild-type strain (SSB1002) in both LB and defined SMS medium at 37°C (Fig. S2A). Western blotting of total extracts showed that the levels of the RNase Y-sfGFP fusion protein were very similar to that of the wild-type RNase Y (Fig. 1B). We imaged mid-log-phase cells grown in SMS medium at 37°C in bright-field, epifluorescence, and TIRF illumination modes (Fig. 1D). RNase Y-sfGFP localized along the cell membrane (Fig. 1D, Epi), forming discrete irregular foci in TIRF mode very similar to the pattern that we had previously observed for the ectopically expressed RNase Y-GFP (compare Fig. 1C and D). A time series streaming acquisition of 20 to 30 s in TIRF mode using a 100-ms exposure time revealed a very dynamic distribution of short-lived RNase Y foci at the cell membrane (Movie S3). Analysis of changes in fluorescence intensity in live cells during a 20-s period showed that the intensity of individual RNase Y foci fluctuated over 196 consecutive images (Fig. S3). This suggests that RNase Y can diffuse by associating/dissociating from the foci in all directions and without any correlation with the cell axis. The localization of RNase Y in the form of foci tethered to the cell membrane is strictly dependent on the N-terminal transmembrane domain. A strain (SSB2066) expressing exclusively the ΔTMD-RNase Y-sfGFP fusion protein shows a homogenous distribution of RNase Y in the cytoplasm (Fig. S1B).

10.1128/mBio.03337-19.2 FIG S2 Growth and morphology of strains expressing wild-type RNase Y or RNase Y-sfGFP. (A) Wild-type strain SSB1002 and strain SSB2048, expressing the RNase Y-sfGFP fusion protein from the native locus, were grown at 37°C in LB (continuous lines) and defined SMS medium (dashed lines). (B) Phase-contrast microscopy images of individual cells from strains SSB1002 and SSB2048 grown in LB and SMS medium. Samples were taken in mid-exponential and early stationary growth phase. Download FIG S2, PDF file, 0.9 MB. Copyright © 2020 Hamouche et al. 2020 Hamouche et al. This content is distributed under the terms of the Creative Commons Attribution 4.0 International license.

10.1128/mBio.03337-19.3 FIG S3 Plot profiles of RNase Y-sfGFP focus distribution and intensity on the membrane over 196 consecutive Z-stacks. Fluorescence intensities were monitored over the long axis of the cell (yellow line) in live mode. Measurements were extracted from the streaming video (ImageJ) and plotted with respect to the position along the long axis of the cell (∼4 μm). Profiles corresponding to Z-stacks 1, 32, 64, 96, 128, 160, and 196 are shown. Fluorescence intensity decreases toward deeper stacks because of photobleaching during acquisition. Download FIG S3, PDF file, 0.1 MB. Copyright © 2020 Hamouche et al. 2020 Hamouche et al. This content is distributed under the terms of the Creative Commons Attribution 4.0 International license.

10.1128/mBio.03337-19.9 MOVIE S3 TIRF time lapse acquisition of RNase Y-sfGFP in live cells (strain SSB2048, mid-log phase in SMS minimal medium at 37°C) in continuous illumination mode (duration, 30 s; exposure time, 100 ms). RNase Y-sfGFP forms very dynamic fast-moving foci at the cell periphery. Download Movie S3, AVI file, 3.9 MB. Copyright © 2020 Hamouche et al. 2020 Hamouche et al. This content is distributed under the terms of the Creative Commons Attribution 4.0 International license.

### RNA substrate availability does not affect RNase Y dynamics.

**FIG 2:**
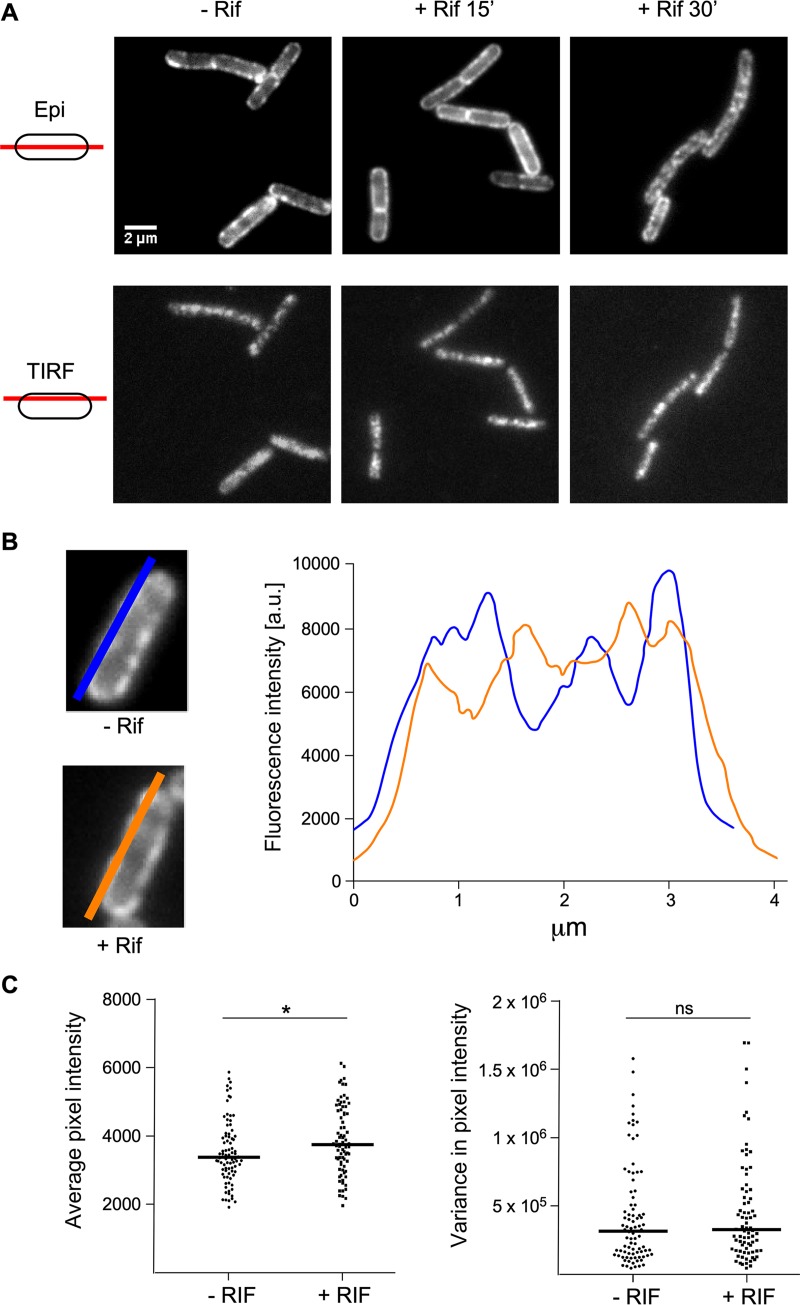
Transcription arrest does not alter RNase Y distribution at the membrane. (A) Strain SSB2048 (RNase Y-sfGFP expressed from native locus) was grown to mid-log phase in SMS minimal medium at 37°C. (Upper) Gallery of epifluorescence micrographs; (lower) images derived from 20-s time-lapse videos recorded in TIRF mode (100 ms). The images show the localization of RNase Y-sfGFP before and after treatment with rifampin for 15 min and 30 min. The schemes on the left side illustrate the focus area. (B) Distribution and fluorescence intensity of RNase Y-SFGFP on the membrane before and after treatment with rifampin. The blue and orange lines drawn over the long axis of the cells schematize the traces that were used to scan the fluorescence intensities. Only values measured within the membrane region were taken into account for quantification. The graph depicts fluorescence intensity as a function of the position along the entire length of the cell (∼4 μm). a.u., arbitrary units. (C) Statistical analysis of a field of cells before and 30 min after treatment with rifampin. Eighty-seven line scans of cells before and 79 line scans of cells after rifampin treatment were analyzed to generate plots of average pixel intensity and variance in pixel intensity. The horizontal line in each plot shows the median. ns, not significant; *, 0.01 < *P* < 0.05.

We next wanted to know whether substrate availability could affect the localization and dynamic behavior of RNase Y. Messenger RNAs are the principal substrates of RNase Y that do not participate in rRNA maturation. To test this possibility, we grew B. subtilis strain SSB2048 in the presence and absence of the transcription inhibitor rifampin. Exponentially growing cells were treated with rifampin, and the dynamic distribution of RNase Y-sfGFP within the cell was examined after 15 and 30 min. We found that the absence of mRNA substrates had no significant effect on the discrete membrane localization and mobility of RNase Y (Fig. 2A, images, and Movie S4). To analyze the distribution and fluorescence intensity profiles of RNase Y at the membrane, we performed line scans over the long axis of the cells (Fig. 2B). The derived plots of average pixel intensity and variance allowed us to quantify changes in the distribution and level of RNase Y on the membrane. We detected no significant differences between rifampin-treated and nontreated cells. The distribution of fluorescence intensity as well as its variance over the entire cell length were very similar (Fig. 2C). We conclude that the availability of mRNA substrates is not a major factor that determines the distribution of RNase Y foci in B. subtilis.

10.1128/mBio.03337-19.10 MOVIE S4 TIRF time lapse acquisition of RNase Y-sfGFP in live cells (strain SSB2048, mid-log phase in SMS minimal medium at 37°C) in continuous illumination mode (duration, 20 s; exposure time, 100 ms) after 30 min of treatment with rifampin (150 μg/ml). Download Movie S4, AVI file, 1.3 MB. Copyright © 2020 Hamouche et al. 2020 Hamouche et al. This content is distributed under the terms of the Creative Commons Attribution 4.0 International license.

### Quantitative analysis of RNase Y focus dynamics.

**FIG 3:**
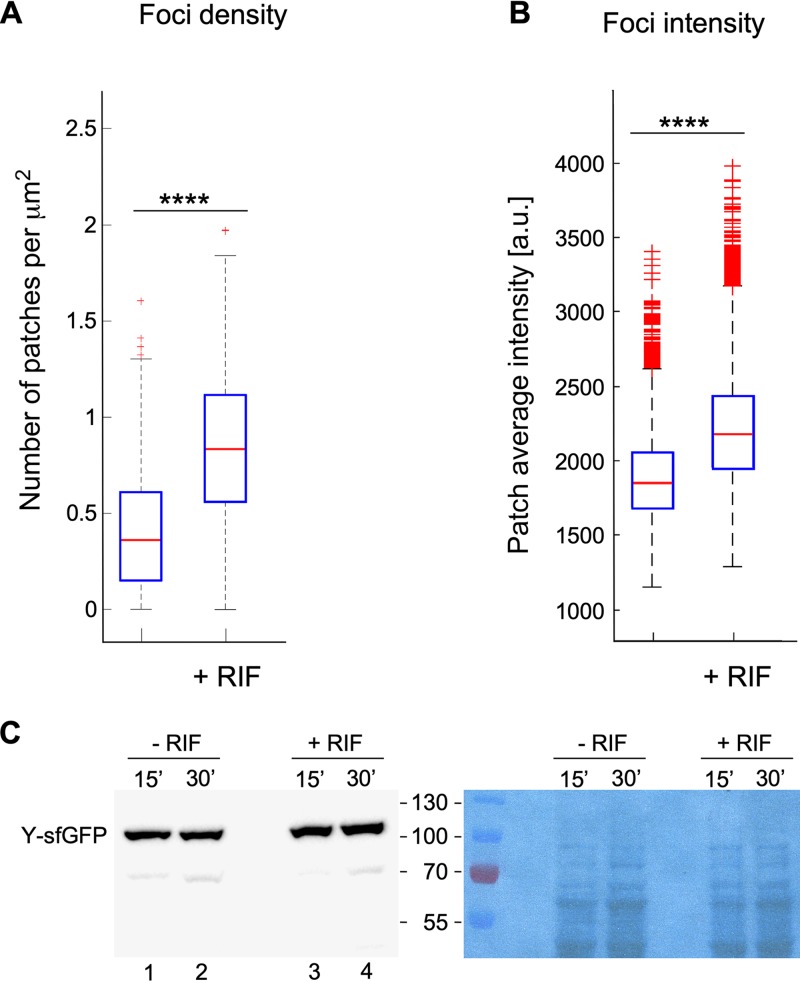
RNase Y focus formation and intensity are increased upon transcription arrest. Formation of RNase Y-sfGFP foci in B. subtilis SSB2048 cells growing exponentially in SMS medium was quantified before and 30 min after transcription arrest (addition of rifampin). (A) Density of RNase Y foci. (B) Intensity of RNase Y foci. The blue boxes represent the interquartile range (25th to 75th percentiles), the red line in the box is the 50th percentile (median), and the black dotted bars represent the range of results, excluding outliers. The red plus signs are outliers. Statistical significance was calculated using the nonparametric Mann-Whitney test (****, *P < *0.0001; ***, 0.0001 < *P* < 0.001; **, 0.001 < *P* < 0.01; *, 0.01 < *P* < 0.05; ns, *P* > 0.05). (C) Quantification of RNase Y-sfGFP expression by Western blotting following transcription arrest. Total cell extracts from exponentially growing cultures (LB medium) of strain SSB2048 with (15’ or 30’) or without addition of rifampin were run on 10% SDS polyacrylamide gels, and RNase Y was detected and quantified using an anti-RNase Y monoclonal antibody. The RNase Y-sfGFP fusion protein (87 kDa) contains the sfGFP polypeptide (28 kDa) fused to the C-terminal end of RNase Y. The times indicated for the cultures grown in the absence of rifampin simply indicate that the samples were taken at the same time as the rifampin-treated cultures. The right panel is a loading control and shows the amido black-stained membrane prior to incubation with the antibody.

The time lapse streaming analyses of RNase Y localization by simple visualization described above suggested that the distribution of RNase Y is not affected by the availability of its substrates. We then evaluated whether the availability of RNA substrate influences RNase Y focus density, i.e., the number of RNase Y foci per cell surface area. The average number of foci identified under TIRF illumination mode was estimated at ∼0.3/μm^2^. Depletion of RNA substrates following addition of rifampin led to an almost 3-fold increase in focus density (∼0.8/μm^2^) (Fig. 3A). Using a linear correlation between the two-dimensional (2D) cell surface area estimated from bright-field images and overall focus density, this corresponds to an increase in RNase Y foci from ∼2 to 5 to 6 per cell in the absence of RNA substrates. In addition, the intensity of the foci, a measure for the relative number of RNase Y molecules in a focus, also increased slightly by ∼22% 30 min after transcription arrest (Fig. 3B). Under these conditions, the absolute amount of RNase Y in the cell increased by about 40% compared to that of untreated cells (Fig. 3C, compare lanes 2 and 4).

We then decided to measure and quantify the RNase Y movements employing single-particle tracking (SPT) tools to analyze at the single-cell level the effect of transcription arrest on the dynamics of RNase Y foci at the membrane (see Fig. S4 and Materials and Methods).

10.1128/mBio.03337-19.4 FIG S4 Organogram illustrating identification of RNase Y foci and their trajectories on TIRF videos. (A, left) Cells are contoured in black. Individual objects (foci) are detected by SPT (single-particle tracking) analysis and attributed a different color, which allows us to trace its complete trajectory independently from other detected objects. (Right) Overview of cell segmentation. Each cell is identified by a random color and designated a number. (B) For a quantitative analysis of RNase Y foci at the single-cell level, time-lapse movies of individual cells (yellow contoured zones) were clipped out of the full-field TIRFm raw movie for further analysis. (C) The major axis was identified for each cell, and its angle to the horizontal axis was calculated. Prior to further analysis, all cells were rotated to the horizontal orientation. (D) Each image corresponds to a different cell (cell 1 in first row and first column, cell 2 in the first row, 2nd column, etc.), using the same numbers as those used for panel A. The randomly colored trajectories of the different objects are plotted. (E) Set of trajectories obtained from the same video. This comprehensive view was obtained by plotting trajectories compiled from the images in panel A without distinguishing individual cells. The scales on the *x* and *y* axes are given in micrometers. Download FIG S4, PDF file, 0.5 MB. Copyright © 2020 Hamouche et al. 2020 Hamouche et al. This content is distributed under the terms of the Creative Commons Attribution 4.0 International license.

**FIG 4:**
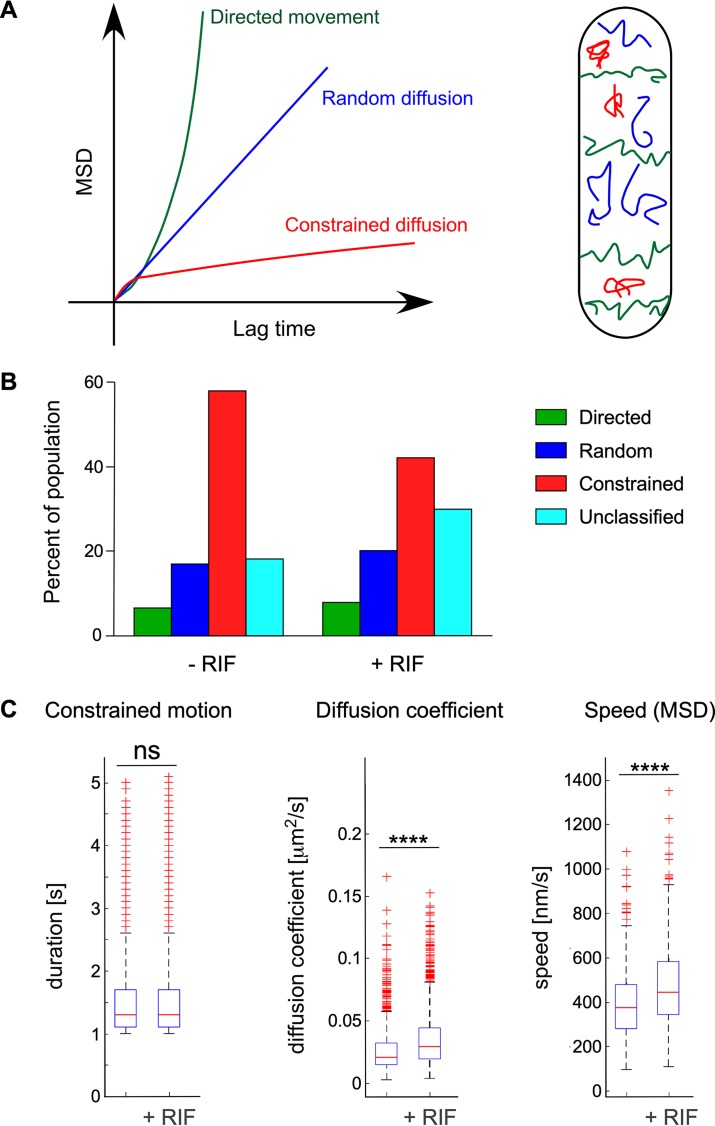
RNase Y focus dynamic behavior and effect of transcription arrest. Strain SSB2048, expressing an RNase Y-sfGFP fusion protein, was grown in SMS medium and analyzed at mid-exponential phase. RNase Y focus motion was categorized based on MSD curve characteristics (as described in the legend for Fig. S6) (45). (A) MSD curves characteristic of directed movement [green curve, MSD(*t*)= (*vt*)^2^], random diffusion [blue curve, MSD(*t*)= 4*Dt*], and constrained diffusion (red curve, MSD reaches a plateau). On the right is a depiction of a rod-shaped cell illustrating the three modes of movement of 2D-SPT trajectories of RNase Y assemblies. (B) Percentage of RNase Y foci that can be categorized into one of the three motion profiles or, if not applicable, into an unclassified population (light blue). Transcription arrest was induced in strain SSB2048 (*rny-sfgfp*) by addition of rifampin to mid-log cells for 30 min. Color code for different types of motion is the same throughout the figure. RNase Y motion was analyzed under normal growth conditions and following transcription arrest (+Rifampin) as described in Materials and Methods. (C) Effect of transcription arrest on the kinetic parameters of RNase Y motion patterns. RNase Y movements were recorded by TIRFm of strain SSB2048 (expressing RNase Y-sfGFP) and categorized after fitting of MSD curves. Strains were analyzed under normal growth conditions and 30 s after transcription arrest. Constrained motion foci were characterized by the time (seconds) of confinement at the same position before measurable movement set in. For randomly diffusing assemblies, a linear fit model was used to calculate the diffusion coefficient (square micrometers per second). For foci belonging to the directed movement category, the average speed was calculated by a second-order polynomial fit. The blue boxes represent the interquartile range (25th to 75th percentiles), the red line in the box indicates the 50th percentile (median), and the black dotted bars represent the range of results, excluding outliers. The red plus signs are outliers. ns, not significant; ****, *P* < 0.0001.

Dynamic behavior of individual foci was determined by mean square displacement (MSD) analysis of their trajectory (Fig. 4A) and used to compile average MSD (45). The highly dynamic foci exhibited different types of motion (several MSD curves) (Fig. 4A). Observing >18,000 RNase Y focus trajectories under each condition and strain, we found that in the wild-type strain (SSB2048) under normal growth conditions, a majority (almost 60%) of RNase Y foci appeared constrained, while ∼25% displayed directed or random motions (Fig. 4B). Unclassified motions correspond to trajectories (<20%) that can be referred to as blended types of motion (45). Interestingly, upon transcription arrest (30 min after addition of rifampin), the proportion of the constrained fraction decreases to ∼40%, in favor of the unclassified and, to a lesser extent, the random motion category (Fig. 4B), indicating a decreased number of static (constrained) foci.

10.1128/mBio.03337-19.5 FIG S5 Principle of mean square displacement (MSD) analysis. (A) Individual MSD curves were calculated and plotted as a function of time (in seconds) for each detected object. (B) From these data, an average MSD was calculated (black line), with the error bars indicating the standard deviation and the light gray zone corresponding to the weighted standard deviation over all MSD curves. (C) The mean of the quadratic displacements, *r*^2^ (*r*^2^_1_ during 1 image, *r*^2^_2_ during two images, etc.), was measured for increasing time intervals and plotted with respect to the time intervals. The example shows a random diffusion trajectory: the curve, *r*^2^(*t*), is a straight line whose slope is proportional to the diffusion coefficient. Download FIG S5, PDF file, 0.2 MB. Copyright © 2020 Hamouche et al. 2020 Hamouche et al. This content is distributed under the terms of the Creative Commons Attribution 4.0 International license.

10.1128/mBio.03337-19.6 FIG S6 Effect of Y-complex mutations and substrate overproduction on the kinetic parameters of RNase Y motion patterns. RNase Y movements were recorded by TIRFm, and motion patterns were categorized after fitting of MSD curves. (A) Effect of Y-complex mutations on RNase Y dynamics. Strain SSB2048 (expressing RNase Y-sfGFP) is presented as the WT. Strains SSB2068 (Δ*ymcA*), SSB2069 (Δ*ylbF*), SSB2070 (Δ*yaaT*), and SSB2075 (Δ*ylbF ΔyaaT*) are derived from SSB2048. Strains were grown in SMS medium. Constrained motion foci were characterized by the time (in seconds) of confinement at the same position before measurable movement set in. For randomly diffusing foci, a linear fit model was used to calculate the diffusion coefficient (in square micrometers per second). For foci belonging to the directed movement category, the average speed was calculated by a second-order polynomial fit. The blue boxes represent the interquartile range (25th to 75th percentiles), the red line in the box indicates the 50th percentile (median), and the black dotted bars represent the range of results, excluding outliers. The red plus signs are outliers. Total number of cells analyzed were 538 cells for SSB2048, 390 cells for SSB2068, 229 cells for SSB2069, 382 cells for SSB2070, and 284 cells for SSB2075. (B) Effect of overexpression of the *yitJ* 5′ UTR on RNase Y dynamics following depletion of endogenous RNA after rifampin treatment. Strains SSB2057 and SSB2058, harboring a chromosomal copy of a xylose-inducible rifampin-resistant T7 RNA polymerase gene, contained a replicative plasmid containing the *yitJ* substrate under control of the T7 promoter (SSB2057) or the empty vector (SSB2058). Addition of xylose 10 min prior to rifampin treatment allowed overexpression of the *yitJ* riboswitch substrate RNA by T7 RNA polymerase (2-fold compared to wild-type expression levels; data not shown) while blocking endogenous mRNA transcription. The different motion categories were analyzed as described for panel A. A plus sign on top of the blot indicates the addition of rifampin. Download FIG S6, PDF file, 2.3 MB. Copyright © 2020 Hamouche et al. 2020 Hamouche et al. This content is distributed under the terms of the Creative Commons Attribution 4.0 International license.

Based on the MSD analysis of individual trajectories from all observed foci, we determined the kinetic parameters of RNase Y movement at the B. subtilis membrane. The characteristics of the constrained RNase Y foci were not affected by transcription arrest; they remained confined at the same position for an average time of 1.6 s with a median value of 1.3 s before detectable movement occurred (Fig. 4C). We calculated the diffusion coefficient for each of the randomly diffusing foci in single cells and extracted the average diffusion coefficient for the entire relevant population using a linear fit model. The median diffusion coefficient increases slightly from 0.02 to 0.03 μm^2^/s in transcriptionally arrested cells (Fig. 4C). The median RNase Y focus velocities of the directed motion category, calculated over the entire trajectory, revealed speeds of ∼400 nm/s that also increased by ∼20% upon transcription arrest (Fig. 4C).

### Effect of YaaT, YmcA, and YlbF on RNase Y dynamics.

**FIG 5:**
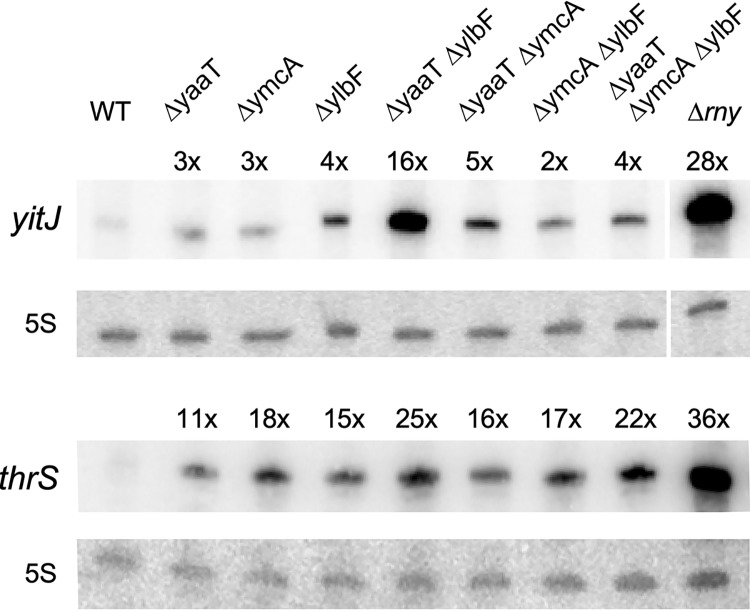
Effect of Y-complex mutations on the cellular levels of *yitJ* SAM riboswitch RNA and the *thrS* leader mRNA. Northern blot analysis of the prematurely terminated *yitJ* and *thrS* 5′ riboswitch RNAs (201 nt and 280 nt, respectively). Total RNA isolated from the indicated strains grown to mid-exponential phase in LB medium was separated on a denaturing 8% acrylamide gel and hybridized to specific continuously labeled RNA probes as described in Materials and Methods. The following strains were analyzed: SSB1002 (WT), SSB576 (Δ*yaaT*), SSB577 (Δ*ymcA*), SSB578 (Δ*ylbF*), SSB579 (Δ*yaaT ΔylbF*), SSB580 (Δ*yaaT ΔymcA*), SSB581 (Δ*ymcA ΔylbF*), SSB582 (*ΔyaaT ΔymcA ΔylbF*), and SSB503 (*Δrny*). Hybridization to 5S RNA was used as a loading control.

A complex of YaaT, YlbF, and YmcA (the Y-complex), previously investigated as a regulator of biofilm formation (31, 37), was recently shown to physically interact with RNase Y and play an important role in the maturation of polycistronic mRNAs (30, 33). In this study, we analyzed the effect of single, double, and triple mutants of the *yaaT*, *ymcA*, and *ylbF* genes on the cleavage of the riboswitch RNAs *yitJ* and *thrS* localized in the 5′ untranslated regions (UTRs) of the respective genes. Both RNAs were stabilized in the mutants, unlike that of the WT strain. The *yitJ* riboswitch level increased 28-fold in an RNase Y null mutant and was still increased up to 16-fold in the Δ*yaaT ΔylbF* double mutant (strain SSB579) and 2- to 5-fold in all other mutants (Fig. 5). Y-complex mutations had a similar effect on the abundance of the *thrS* riboswitch, where the strongest stabilization was also observed in the Δ*yaaT ΔylbF* double mutant (Fig. 5).

**FIG 6:**
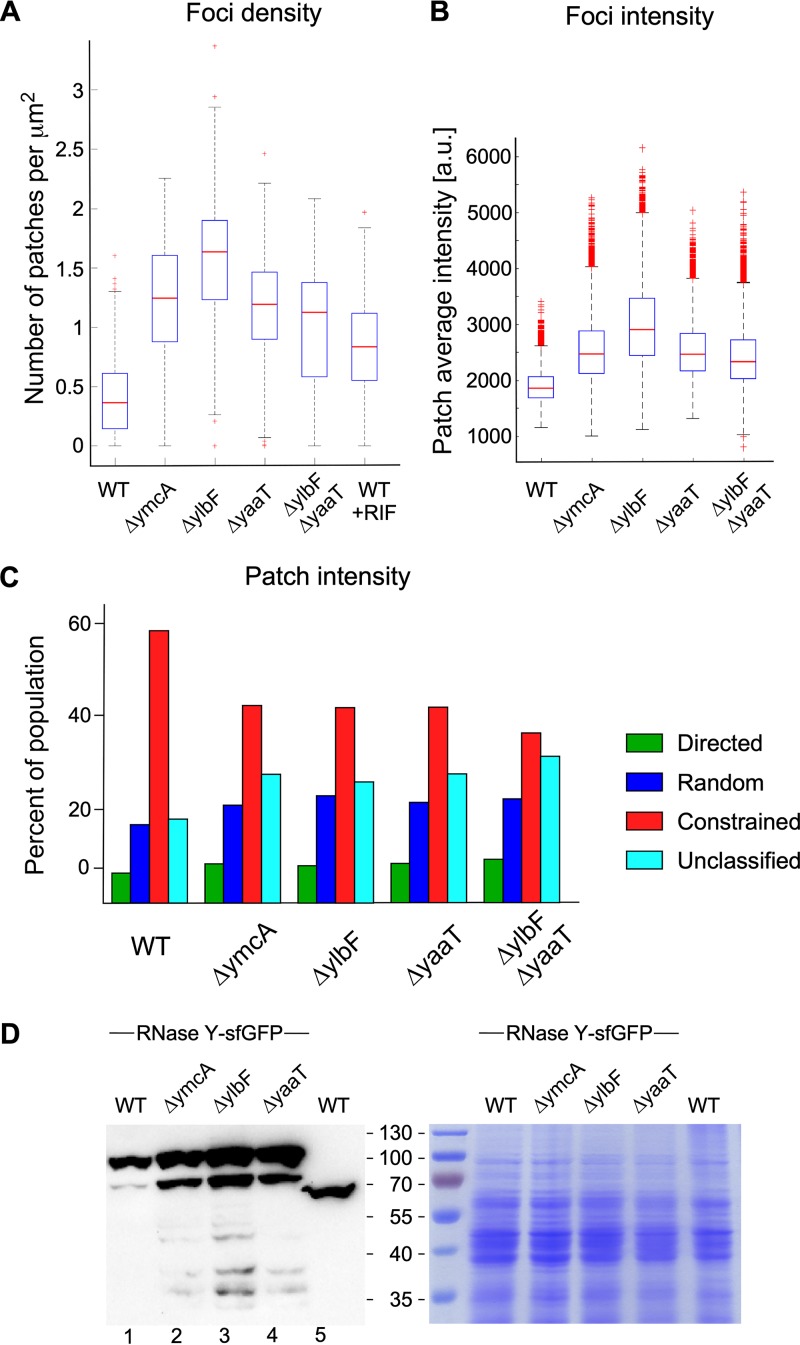
Y-complex mutations affect the dynamics, number, and size of RNase Y foci and induce RNase Y expression. All strains tested express RNase Y-sfGFP. Strain SSB2048 (WT), SSB2068 (*ΔymcA*), SSB2069 (*ΔylbF*), SSB2070 (*ΔyaaT*), and SSB2075 (*ΔylbF ΔyaaT*) were grown to mid-exponential phase in SMS medium and analyzed by TIRFm as described in Materials and Methods. (A) Density of RNase Y foci calculated at the single-cell level. (B) Total intensity distribution of RNase Y foci calculated at the single-cell level. Data from the three independent experiments were averaged, totaling 538 cells for SSB2048, 390 cells for SSB2068, 229 cells for SSB2069, 382 cells for SSB2070, and 284 cells for SSB2075. The blue boxes represent the interquartile range (25th to 75th percentiles), the red line in the box is the median, and the black dotted error bars correspond to the standard deviations, excluding outliers. Red plus signs represent the outliers. Nonparametric Mann-Whitney test was used for statistical analysis (****, *P* < 0.0001; ***, 0.0001 < *P* < 0.001; **, 0.001 < *P* < 0.01; *, 0.01 < *P* < 0.05; ns, *P* > 0.05). (C) Percentage of RNase Y foci that can be categorized into one of the three motion profiles or, if not applicable, into an unclassified population (see also Fig. 4). (D) Quantification of RNase Y-sfGFP and RNase Y expression by Western blotting. Total cell extracts from exponentially growing cultures (LB medium) of the indicated strains were run on 12% SDS polyacrylamide gels, and RNase Y was detected and quantified using an anti-RNase Y monoclonal antibody. Strain SSB1002 expresses wild-type RNase Y (58.9 kDa), while the other strains express the RNase Y-sfGFP fusion protein (87 kDa), which contains the sfGFP polypeptide (28 kDa) fused to the C-terminal end of RNase Y.

The Y-complex has been proposed to function as a specificity factor rather than a general activator of RNase Y activity (33), but it is not clear how this might be achieved. We first studied whether Y-complex mutations could alter the size and number of RNase Y foci at the membrane. A strong increase in focus density was observed in the mutant strains. The number of RNase Y foci/μm^2^ was 3.5× to 4.5× higher in the mutant strains, with the strongest effect observed in the *ΔylbF* strain (Fig. 6A). We also measured a significant increase in focus intensity (1.5× to 2×) for all mutants compared to the level for the wild-type strain, with the *ΔylbF* strain again showing the greatest effect (Fig. 6B). The strongly increased number of RNase Y foci at the membrane, coupled with the increased size of the foci (focus intensity), suggested that RNase Y expression itself is induced in the Y-complex mutant strains. We therefore determined RNase Y levels by Western blotting in total cell extracts. The Y-complex single mutations led to a 1.6-, 2-, and 2-fold increase in RNase Y in the *ΔymcA*, *ΔylbF*, and *ΔyaaT* strains, respectively, compared to that of the wild-type strain (Fig. 6D). This increase is even stronger (up to 3-fold) if we also consider the accumulation of a likely degradation product with an apparent molecular weight (MW) of ∼80 kDa (Fig. 6D).

We next analyzed whether the Y-complex has the potential to affect the dynamics of RNase Y movement. Single-knockout mutations of the three Y proteins and a *ΔyaaT ΔylbF* double mutation were introduced into strain SSB2048, expressing the RNase Y-sfGFP fusion protein from the native locus. We characterized the effect of these mutations on the three categories of RNase Y motion (directed, random, and constrained) that we had identified by the MSD analysis described above. In all mutants, we observed a significant decrease of the constrained motion population from 60% (WT) to ∼38% in the *ΔyaaT ΔylbF* double mutant, which showed a slightly stronger effect than the three single-mutant strains (Fig. 6C). This reduction of constrained RNase Y foci was compensated by an increase in the random motion and unclassified categories, with the directed motion class remaining constant in all strains (Fig. 6C).

The analysis of RNase Y focus kinetics revealed no significant differences between the wild-type and mutant strains. The time of confinement for the constrained foci was 1.3 s in the wild type and increased by at most 0.2 s in the mutant strains (Fig. S6A). The diffusion coefficient of the random motion category remained constant at ∼0.02 μm^2^/s, and the speed of the directed motion foci was stable at ∼400 nm/s (Fig. S6A).

## DISCUSSION

A major observation from this work is evidence that RNase Y forms highly motile foci along the cell membrane, whose dynamics can be altered by substrate availability and interaction with protein partners. In accordance with the presence of an N-terminal transmembrane domain, RNase Y has been reported to localize to the periphery of the cell and to form foci at the division septum (11, 12). Epifluorescence microscopy, carried out in this study under live cell conditions, confirmed this localization. However, only under TIRF illumination mode could we observe that RNase Y actually forms discrete assemblies (foci) that are present on the entire cell surface. They are short-lived and diffuse rapidly over the entire membrane. Moreover, the fluorescence intensity of the RNase Y foci fluctuates appreciably between consecutive images and across a significant depth of field. Such fluctuations are not observed for membrane-tethered versions of GFP alone (46), indicating that the foci are dynamic structures where RNase Y-sfGFP molecules freely associate/dissociate. Since RNase Y carries an N-terminal transmembrane domain, this redistribution likely occurs entirely between membrane-tethered proteins. Indeed, we showed that without the transmembrane domain, RNase Y localizes uniformly to the cytoplasm. This strongly suggests that the RNase Y-GFP fusion protein used in this work is not prone to spontaneous self-aggregation, potentially affecting subcellular localization, as has been reported in some cases (41). It also indicates that the membrane anchoring of RNase Y is not vital for B. subtilis, as reported previously (47).

Our observations of RNase Y dynamics are reminiscent of the model proposed for E. coli RNase E, in which formation of membrane-tethered foci and constraints on diffusion arise from the transient clustering of RNase E into cooperative degradation bodies (10). However, here we present compelling evidence that the apparent analogy between RNase E and RNase Y membrane dynamics does not translate into functional equivalence. Indeed, depleting the cell of RNA substrates through transcription arrest does not reduce or smooth out the patchy pattern of RNase Y distribution at the membrane. To the contrary, following addition of rifampin the number of RNase Y foci doubled, and their intensity increased by >20%. This clearly suggests that clustering of RNase Y in membrane-anchored foci represents an inactive, or less active, form of the enzyme. It also indicates that RNase Y is a stable protein whose quantity is not significantly affected by substrate availability. Single-particle tracking tools and a mean square displacement (MSD) analysis indicated that in exponentially growing cells, 60% of RNase Y foci exhibit a constrained diffusion behavior, i.e., they remain immobile for more than 1 s. We propose that the constrained motion foci are the principle active forms of RNase Y and that the reduced mobility is caused by the interaction with an RNA substrate. Indeed, in B. subtilis translation occurs predominantly around the periphery of the cell (13), where RNase Y would encounter actively translated polycistronic substrates to be processed or a suboptimally translated mRNA to initiate its degradation. In agreement with this hypothesis, the number of foci increases 2-fold following transcription arrest, but the share of constrained foci drops from 60% to 40%. The remaining 40% of constrained foci may be due to an only partial depletion of RNA from the cell, as the analysis was carried out 30 min after addition of rifampin. This assumption would also fit with the observation that the interval during which movement of the constrained foci is restricted remains constant under all conditions at ∼1.3 s. The diffusion rate of randomly diffusing assemblies and the speed of directed motion foci were slightly increased following transcription arrest. It is noteworthy that the measured translation speed of the directed motion foci attains ∼400 nm/s, which is considerably faster than what has been described for other focus-forming membrane proteins, like B. subtilis MreB (∼60 nm/s) (45), suggesting a largely unimpaired freedom of movement.

We could not reverse the effect of transcription arrest on RNase Y focus formation and dynamics when simultaneously overproducing the *yitJ* riboswitch from a T7 promoter (Fig. S6B). However, *yitJ* RNA synthesis under these conditions was only about 2-fold higher than normal wild-type levels and presumably could not compensate for the loss of global cellular RNA substrates following addition of rifampin.

Interestingly, knockout mutations of the Y-complex genes *yaaT*, *ylbF*, and *ymcA* have impact on RNase Y dynamics similar to, albeit much stronger than, that of depletion of RNA substrates, keeping in mind that rifampin treatment probably does not lead to a complete removal of mRNA in the cell. Individual gene deletions of proteins from the Y complex increased the number of RNase Y foci up to 4.5-fold and their intensity by about 2-fold. This increase in both the number and relative size of the foci can at least partially be explained by the ∼2-fold increased concentration of RNase Y under these conditions. However, RNase Y clearly has a propensity to form higher-molecular-weight assemblies at the membrane. The individual Y-complex mutations have been shown to reduce cleavage/processing of only a relatively small proportion of RNase Y substrates, essentially polycistronic mRNAs and, to a lesser degree, riboswitches (33). The *rny-sfgfp* transcript might also be affected, which would explain the 2- to 3-fold increase in the RNase Y-sfGFP protein level observed in the mutant strains.

The degradation product of ∼80 kDa likely lacks sequences from the C-terminal GFP domain, as the N-terminal part of RNase Y upstream of the antibody recognition site would have an MW of less than 9 kDa, which is too small to account for the apparent difference of ∼20 kDa in MW.

Our data suggest that the Y-complex acts by modulating the size and number of the membrane-associated RNase Y assemblies. Since a higher focus intensity (i.e., size of foci) coincides with a reduced activity toward certain substrates, even when the number of foci (and the intracellular level of RNase Y) is significantly increased, we suggest that smaller enzyme complexes constitute the more active form of RNase Y, at least with respect to RNA substrates whose cleavage depends on the Y-complex. In addition, the assembly of RNase Y complexes appears to occur in a continuum without discrete steps; individual Y-complex mutants as well as the *ylbF-yaaT* double mutant lead to a rather continuous pattern of increase in focus intensity. The simultaneous increase in intensity and number of RNase Y foci is obviously also linked to the observed increase in RNase Y levels. However, the fact that more RNase Y expression leads to less activity in the cell indicates that the observed assembly into larger complexes represents a less active form of the enzyme.

It has recently been shown that the YmcA, YlbF, and YaaT proteins form a ternary complex with a stochiometry of 1:1:1 (32). While individual pulldown experiments suggested that RNase Y can interact with YaaT and YlbF (30, 31), there is no evidence that YmcA or the three proteins together bind to RNase Y. In addition, since YaaT is considerably more abundant than YmcA and YlbF, different associative forms likely exist *in vivo* (32). This could explain the differential effects of the individual mutants observed here. In the case of the *yitJ* and *thrS* riboswitches, both of which depend on the Y-complex for their efficient degradation (33), we found that their levels are highest not in the triple mutant but rather in the *ylbF-yaaT* double mutant. This again relates directly to our observation that the interaction of the Y-complex proteins with RNase Y can influence the global assembly status of RNase Y over a significant range of both number and size of foci (Fig. 6). The Y-complex genes *ymcA*, *ylbF*, and *yaaT* are transcribed at similar levels during growth and turned off as the culture enters stationary phase. However, only *ymcA* and *ylbF* depend on Spo0A for their downregulation, suggesting a complex regulatory mechanism (31). In addition, it will be important to explore whether the Y-complex serves as a metabolic or environmental sensor of mRNA stability in light of recent evidence that the Y-complex carries iron-sulfur clusters (39) and that these clusters appear essential for RNA maturation (32).

**FIG 7:**
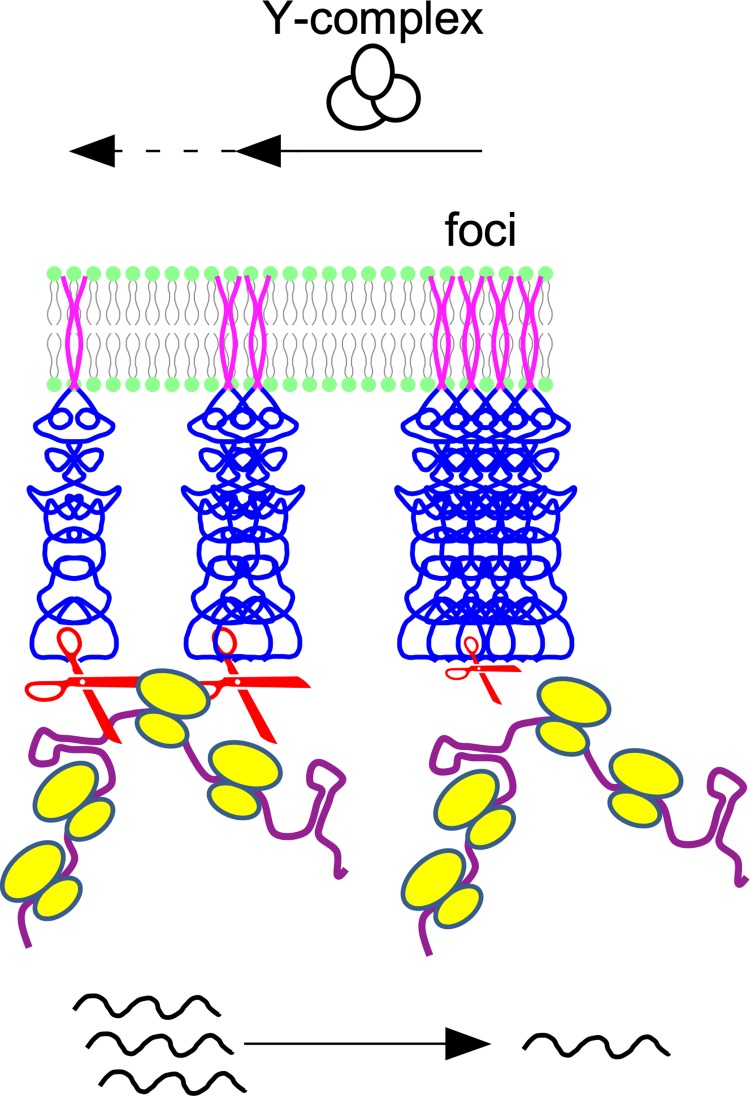
Model of the role of the Y-complex and substrate availability on the RNase Y assembly state. In the presence of the Y-complex or its individual proteins, the large default RNase Y assemblies decrease in number and size. This increases the number of smaller RNase Y complexes, which can sterically access bulky substrates (e.g., translated polycistronic mRNAs) more easily or simply faster due to the more efficient surface distribution of active enzyme. In the absence of RNA substrates the freed smaller RNase Y complexes tend to form higher-order assemblies by default.

Since the Y-complex mainly affects maturation of mRNA in operons without any recognizable sequence or structural specificity (33), our work raises the question of how the assembly status of RNase Y at the membrane can impact RNA cleavage. RNase Y without its transmembrane domain forms mostly elongated dimers in solution (47), and we have shown that this form of the enzyme is active *in vitro* (17). The formation of dimers is likely induced by the extensive unstructured N-terminal domain (∼200 amino acids), which alone can dimerize while undergoing conformational changes (48). In TIRF microscopy, the RNase Y dimers are not resolved because they are shorter than the diffraction limit. Moreover, most of the RNase Y molecules appear to be present in the form of very bright foci. The resulting shorter exposure times probably render dimeric RNase Y molecules practically invisible. These large RNase Y assemblies most likely are also active in cleaving RNA, notably the subpopulation that shows the constrained motion profile. At present, we cannot estimate how many RNase Y molecules are present in an average focus, but for unknown reasons they could be less efficient in cleaving the most bulky substrates, like translated polycistronic mRNAs. At the same time, assembling RNase Y into a few large structures should inevitably reduce the availability of isolated low-molecular-weight forms of the enzyme, which should be more evenly distributed along the membrane, as suggested by the epifluorescence images (Fig. 1). Their reduction could, for example, reduce the rapid access of abundantly transcribed riboswitches to RNase Y for degradation. In our model, summarized in Fig. 7, we view the Y-complex and its individual components as a subtle chaperon-like modulator of RNase Y activity by shifting the equilibrium from higher- to lower-molecular-weight assemblies of RNase Y. However, despite some preliminary data that Y-complex proteins do not alter ΔTMD-RNase Y activity and specificity *in vitro*, we also cannot exclude the possibility that these proteins play a more direct role in influencing the specificity of membrane-bound RNase Y *in vivo.*

The extent to which the addition of the rigid GFP moiety to the C terminus of RNase Y influenced our measurements remains an open question. Since deletion of the transmembrane domain was sufficient to uniformly distribute the RNase Y-GFP fusion protein in the cytoplasm, it is unlikely that the GFP moiety significantly contributes to focus formation. The observed very fast focus velocities of the fusion protein, exceeding 400 nm/s, also suggest that the GFP domain does not strongly interfere with this freedom of movement. Thus, while the qualitative results are likely independent of the presence of the GFP domain, alterations of quantitative aspects might well occur.

An important outstanding question concerns the stoichiometry, structure, and composition of RNase Y foci. This will require superresolution microscopy techniques and *in situ* hybridization approaches to colocalize potential interaction partners, be they membrane tethered or cytoplasmic. Many other factors have been proposed to interact with RNase Y, but these interactions appear to be transient at best (28). However, they may well be sufficient to transiently shift the assembly status of RNase Y under specific conditions, i.e., by interacting with the intrinsically unstructured N-terminal domain of RNase Y. Such domains are promiscuous binders that are constantly involved in various interactions with diverse partners (49). In this respect, it is noteworthy that the N-terminal domain, which contributes to the dimerization and elongated structure of RNase Y, can be shifted to the monomeric state upon interaction with an antibody recognizing a short peptide within the domain (48), suggesting that other factors modulate RNase Y tertiary complexes. Studying these interactions will help us understand the ever-increasing complexity of RNA metabolism in bacteria that not only involves ingenious types of compartmentalization but also modulation of highly dynamic enzyme assemblies.

## MATERIALS AND METHODS

### Bacterial strains and growth conditions.

Strains used in this study are listed in Table 1. The RNase Y deletion strain SSB503 was constructed by replacing the *rny* open reading frame with that of chloramphenicol acetyltransferase (651 nucleotides [nt]) from the S. aureus plasmid pC194 (50). A final PCR fragment containing the *cat* ORF flanked by ∼700 nt from the *rny* up- and downstream regions was used to replace the *rny* ORF through a double recombination event, selecting for chloramphenicol resistance.

**TABLE 1:** B. subtilis strains used in this study

Strain	Relevant genotype	Reference or source
SSB1002	Wild-type strain	1A2 (BGSC)
SSB503	Δ*rny*::*cat*	This work
SSB576	*ΔyaaT*	This work
SSB577	*ΔymcA*	This work
SSB578	*ΔylbF*	This work
SSB579	Δ*yaaT ΔylbF*	This work
SSB580	Δ*yaaT ΔymcA*	This work
SSB581	Δ*ymcA ΔylbF*	This work
SSB582	Δ*yaaT ΔymcA ΔylbF*	This work
SSB2048	*rny-sfgfp*	This work
SSB2063a	*trpC2 thrC*::*sfp^+^-erm ΔamyE*::*Pxyl-rny-mgfp-mut1*	This work
SSB2063b	*trpC2 thrC*::*sfp^+^-erm ΔamyE*::*Pxyl-rny-gfp-mut1*	This work
SSB2066	*rny*(*Δ4-72*)*-sfgfp*	This work
SSB2068	*rny-sfgfp ΔymcA*	This work
SSB2069	*rny-sfgfp ΔylbF*	This work
SSB2070	*rny-sfgfp ΔyaaT*	This work
SSB2075	*rny-sfgfp ΔyaaT ΔylbF*	This work

Strain SSB2063a was constructed by transforming strain OMG930 (51) with plasmid pSG5908-(A206K). Plasmid pSG5908, kindly provided by J. Errington, expresses RNase Y-GFP and is based on the C-terminal GFPmut1 fusion vector pSG1154 (52). After integration at the *amyE* locus, RNase Y-GFP is expressed from the xylose-inducible promoter (11). Expression of *rny-gfp* was induced by the addition of 60 mM xylose (1%). We have introduced the A206K mutation in gfpmut1 to create the monomeric form of GFP (mGFP), which has been shown to strongly reduce the potential formation of nonphysiological foci often observed for GFP fusion proteins (41). Nevertheless, we also analyzed a strain expressing the unmodified RNase Y-GFPmut1 fusion protein (SSB2063b) and detected no differences in localization and dynamics using TIRF microscopy.

Strain SSB2048 was obtained by replacing the native *rny* gene between the start and stop codons with an *rny-sfgfp* construct that expresses the RNase Y-sfGFP fusion protein from the native locus. It was constructed by markerless allelic replacement using the thermoexcisable plasmid pMAD (53). SSB2048 was the parent strain for the construction of all other strains described below except SSB2066. Markerless allelic replacement with pMAD was also used to delete the *yaaT*, *ylbF*, and *ymcA* ORFs between the respective start and stop codons and to create the various single, double, and triple mutants. The different mutations were introduced into the wild-type strain SSB1002 and strain SSB2048. Mutants were verified by sequencing PCR fragments containing up- and downstream regions of the respective gene deletions. All B. subtilis strains are listed in Table 1. Strain 2066 expresses ΔTMD-RNase Y-sfGFP (lacking amino acids 2 to 24, starting with MR25K) from the native locus. It was constructed by markerless allelic replacement using the thermoexcisable plasmid pMAD (53). Construction of strains SSB2057 and SSB2058 will be described elsewhere. Briefly, both strains contain a xylose-inducible copy of the bacteriophage T7 RNA polymerase gene at the *amyE* locus and either the 5’ UTR (SAM riboswitch) of the *yitJ* gene under the control of the T7 polymerase promoter inserted into the replicative plasmid pDG148 (SSB2057) or the empty vector (SSB2058).

E. coli strains JM109 and XL1-Blue were used for plasmid construction. E. coli was grown at 37°C in LB medium and B. subtilis strains at 37°C in either LB medium or SMS defined medium (Spizizen, 1958) containing 25 mM (NH_4_)_2_SO_4_, 80 mM K_2_HPO_4_, 44 mM KH_2_PO_4_, 3 mM trisodium citrate, 0.8 mM MgSO_4_, fructose 0.75%, ferric ammonium citrate 22 mg/liter, l-glutamine 0.1%, and 0.2 mM l-tryptophan. For threonine auxotrophic strains, liquid and agar media were supplemented with 1 mM threonine. When required, threonine was added at 1 mM, as were the following antibiotics (concentrations): ampicillin (200 μg/ml) for E. coli; chloramphenicol (5 μg/ml), spectinomycin (100 μg/ml), erythromycin (1 μg/ml), lincomycin (12.5 μg/ml), neomycin (7 μg/ml), and tetracycline (10 μg/ml) for B. subtilis. Transcription from inducible Pxyl and Pspac promoters was induced by the addition of 60 mM xylose or 1 mM isopropyl-β-d-thiogalactopyranoside (IPTG), respectively. Transcription arrest was induced by addition of rifampin (150 μg/ml). X-gal (5-bromo-4-chloro-3-indolyl-beta-d-galactopyranoside) and methionine were added as required at 40 μg/ml and 2 mM, respectively.

### Epifluorescence/phase-contrast microscopy.

Overnight cultures of relevant strains in LB or SMS medium were diluted to an optical density at 600 nm (OD_600_) of 0.05 and grown at 37°C in shaking flasks. Samples of 1 to 2 μl were taken in early exponential phase (OD_600_ of 0.2 to 0.4) or in early stationary phase (T2) and spotted on microscope slides coated with a thin 1% (wt/vol) agarose pad and topped with a coverslip. Samples were prepared and imaged at room temperature. Images were acquired with an AxioCam MRm camera (Zeiss) mounted on a Zeiss AxioImager M1 phase-contrast/fluorescence microscope. GFP fluorescent images were taken with a 63× air or a 100× oil objective with a numerical aperture (NA) of 1.3 using filter set 10 (Zeiss). GFP was excited at a wavelength of 450 to 490 nm, and the fluorescence was collected in the range of 515 to 565 nm.

### TIRFm.

Images and time-lapse TIRFm movies were taken on at least two different days for each strain and condition. One microliter of culture spotted on a 1% agarose pad and topped by a cover slide and immersion oil was immediately mounted and analyzed in the temperature-controlled microscope stage at 37°C. Imaging was carried out on an inverted microscope Nikon Ti-E fitted with a 100× oil objective Apo TIRF (NA, 1.49; Nikon) with a diode-pumped solid-state laser (50 mW, 491 nm; Cobolt Calypso) or an iLas2 laser coupling system from Roper Scientific (150 mW, 488 nm). Conventional epifluorescence images were snapshotted with a phase-contrast and GFP fluorescence channel (with an excitation filter wavelength of 472/30 nm and emission filter wavelength of 520/35 nm). The exposure time was set up to 100 ms for 20 s or 30 s of imaging time in continuous illumination mode. All images and time-lapse streaming were collected with an EMCCD (electron-multiplying charge-coupled device) camera (iXON3 DU-897; Andor), with a gain defined at 300 attached to a ×2.5 magnification lens. Final pixel size was 64 nm. Image acquisition was controlled and processed with the Nikon NIS-Elements or Metamorph v.7 software packages. The angles of incidence of the laser beam and z-position were fixed individually for all channels to get an epifluorescence or TIRF illumination and to maintain the same depth of evanescent wave penetration as well as the focus position in all experiments.

### Cell segmentation.

Single-cell analysis required the segmentation of bacteria observed by TIRF microscopy. For that purpose, bright-field images were captured at the same field of view and thresholded to provide binary masks revealing single cells as separate regions of interest (ROIs). Incomplete cells located on the edge of the image were removed from the analysis. The area of the segmented cells has been quantified as the product of the number of pixels of ROI and the pixel area in the image (64 by 64 nm^2^). Cell segmentation was performed using Fiji (54).

### Detection and tracking of RNase Y foci.

Single-particle tracking (SPT) is an image analysis method to detect some labeled objects (and extract their spatial coordinates, *x* and *y*) and follow them over time (*t*) to get their trajectories (*x*, *y*, and *t*). Detection aims to segment and accurately localize objects in images by taking into account their main features (e.g., shape, size, intensity, signal-to-noise ratio, etc.). Trajectories (*x*, *y*, and *t*) are estimated by linking detected objects over time by considering their dynamic properties (maximal distance, diffusion coefficient, and reconnection after missed detection).

Trajectories were generated using the TrackMate (55) plugin in Fiji after a careful optimization of SPT parameters under various conditions. RNase Y, which mainly appears as diffraction-limited objects, was detected with the Laplacian of Gaussians (LoG) detector (estimated blob diameter, 0.3 μm; threshold, 200; perform subpixel localization, no median filter, and no filter on object identification). Tracks were generated using the Simple LAP Tracker, with a 0.3-μm linking max distance and no frame gaps allowed. Finally, tracks were exported into MATLAB for processing (density and intensity of RNase Y, dynamic classification, and quantification).

RNase Y focus density was calculated as the average number of foci detected over all frames in each single cell normalized by its area. Lastly, RNase Y intensity was quantified as the time average of local focus intensity (measured as the mean intensity in a 3- by 3-pixel area centered at detected foci).

### Automatic dynamic classification of RNase Y foci.

RNase Y dynamics were categorized based on MSD analysis, as already described extensively (45) (see Materials and Methods and the supplemental material). Only trajectories with a minimal duration of ten consecutive frames (i.e., 1 s) were considered.

Each RNase Y focus detected was assigned to a class (directed, diffused, constrained, or unclassified) for all frames of its corresponding movie, allowing us to calculate mobile fractions per frame and, thus, to estimate averages at the single-cell level. Speed (ν) was quantified using a solely focus-exhibiting directed motion, and diffusion coefficient was extracted from random diffusion trajectories only.

We have analyzed around 1,000 cells of SSB2048 (with or without rifampin treatment), between 250 and 400 cells of the mutant strains (SSB2068, SSB2069, and SSB2070), and about 280 cells for SSB2075 under each condition (with or without rifampin). We analyzed between 170 and 250 cells for strains SSB2057 and SSB2058 under different conditions (with or without rifampin and with xylose and rifampin).

### Statistical analysis.

GraphPad Prism, version 7.0, was used to carry out nonparametric statistical analysis and tests with two-tailed Mann-Whitney test with an alpha level of 5%.
