# Supplementary figures and images for: Dynamic Membrane Localization of RNase Y in Bacillus subtilis
Source: mBio. 2020 Feb 18;11(1):e03337-19. doi: 10.1128/mBio.03337-19 (PMC7029143; doi:10.1128/mBio.03337-19)

Supplementary Figure 1

A

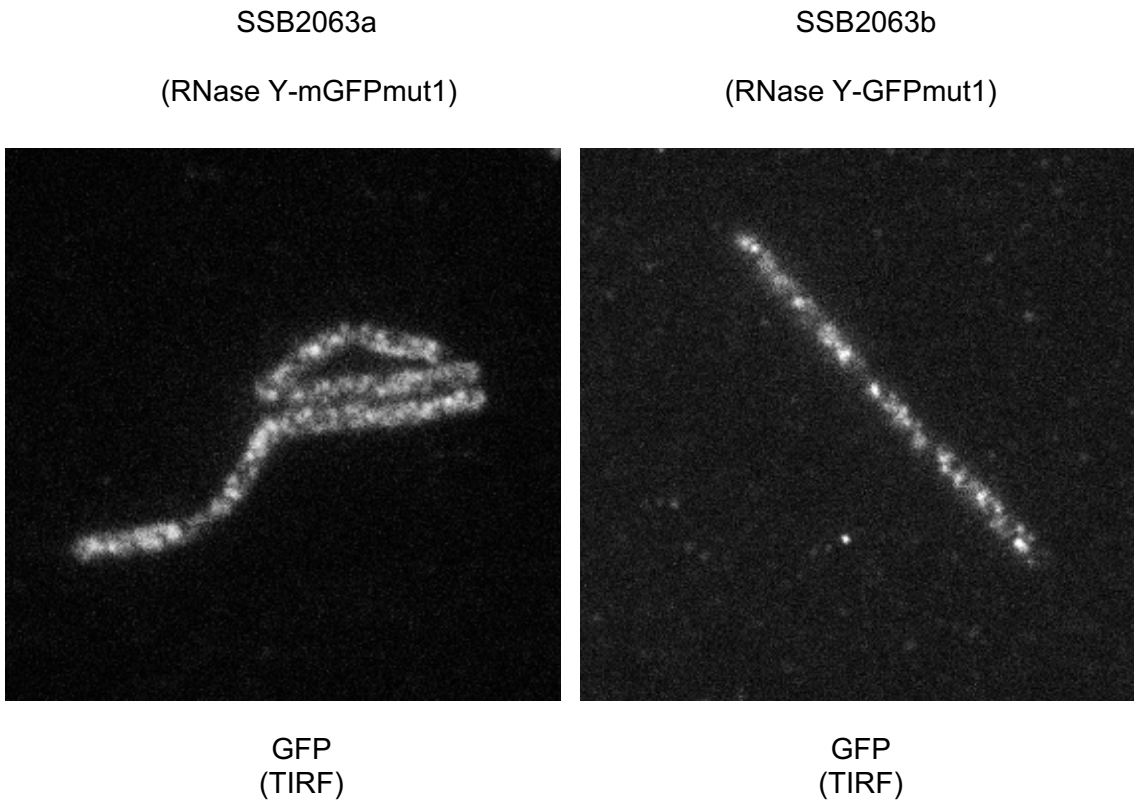

B

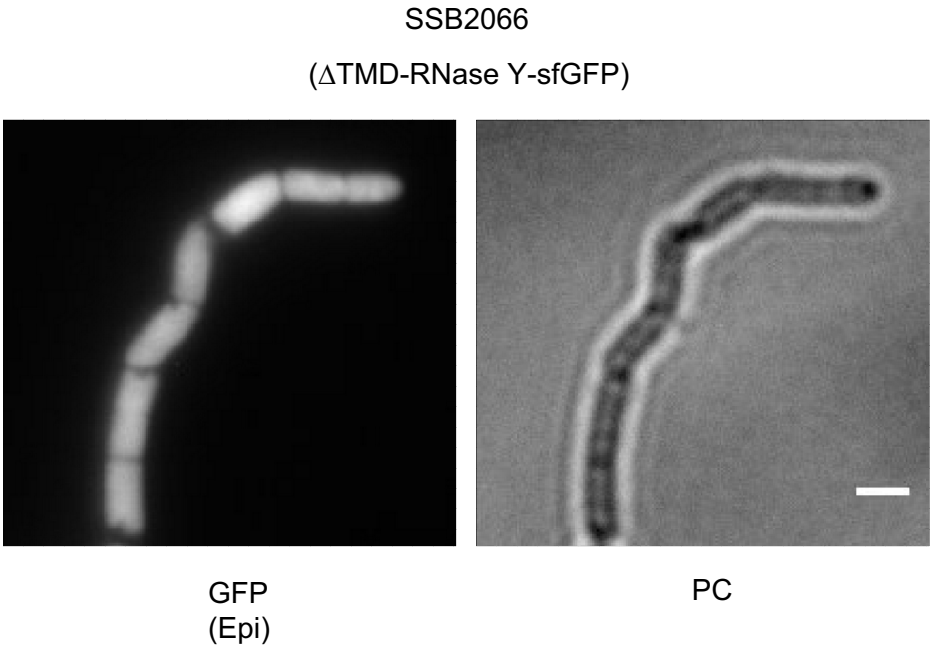

Supplement: FIG S1 [file mBio.03337-19-sf001.pdf]

Supplementary Figure 2

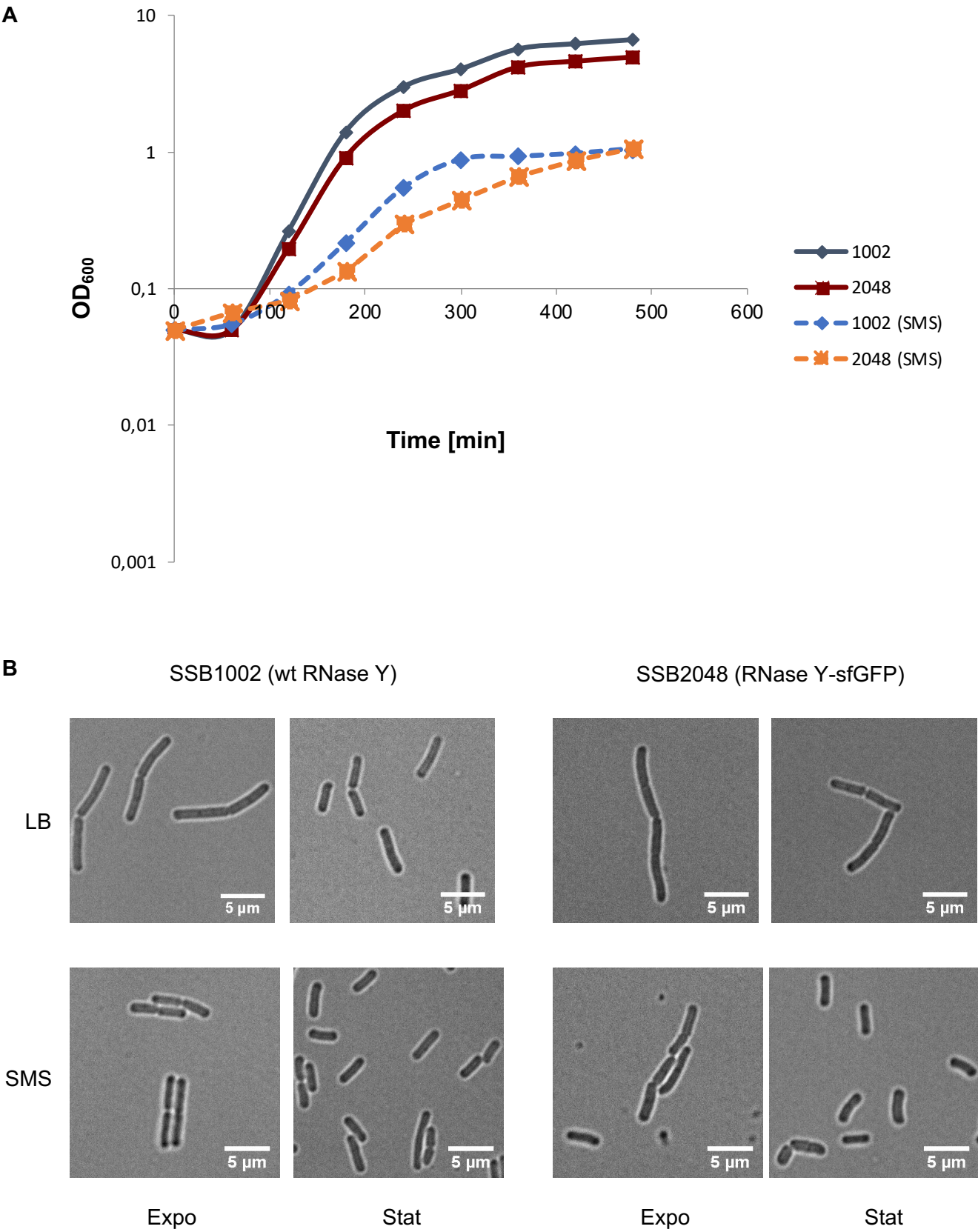

Supplement: FIG S2 [file mBio.03337-19-sf002.pdf]

Supplementary Figure 3

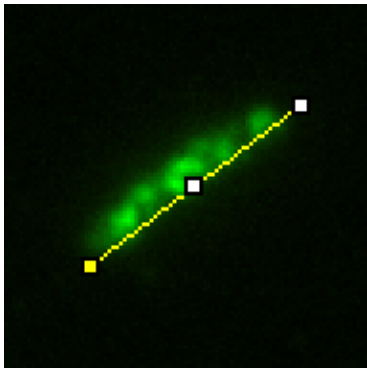

Stack 1

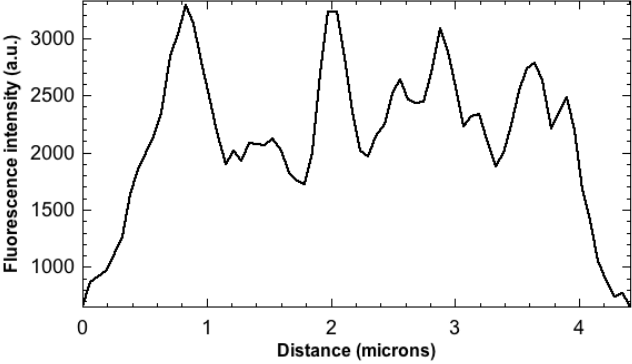

Stack 32

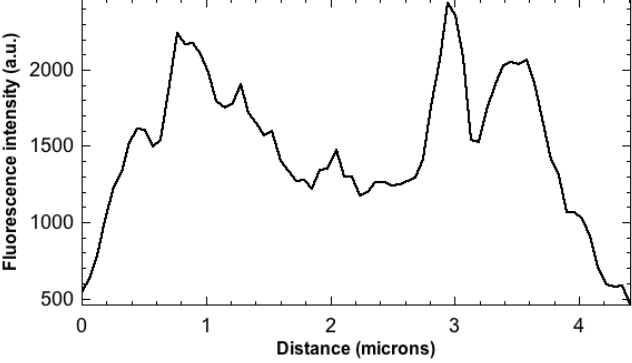

Stack 64

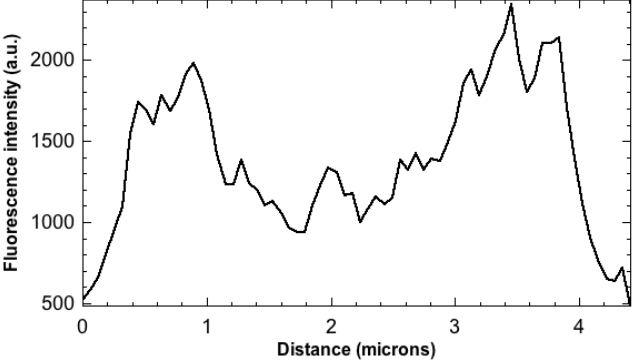

Stack 96

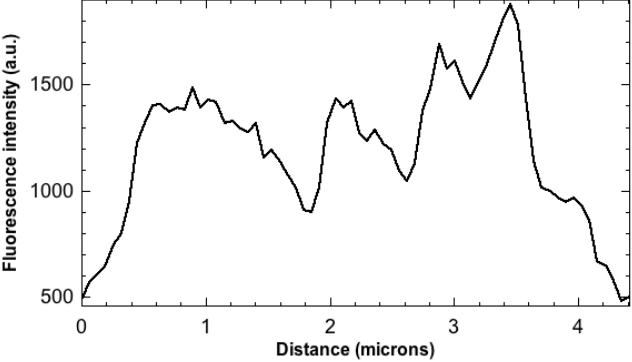

Stack 128

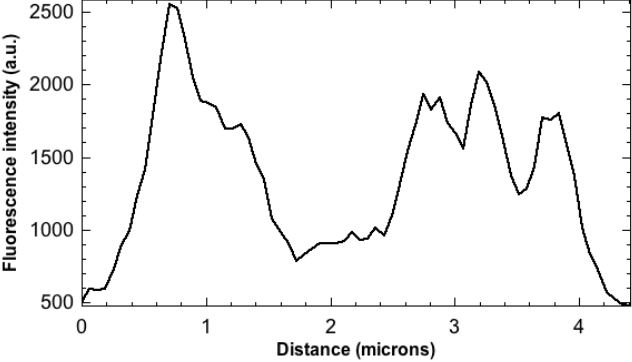

Stack 160

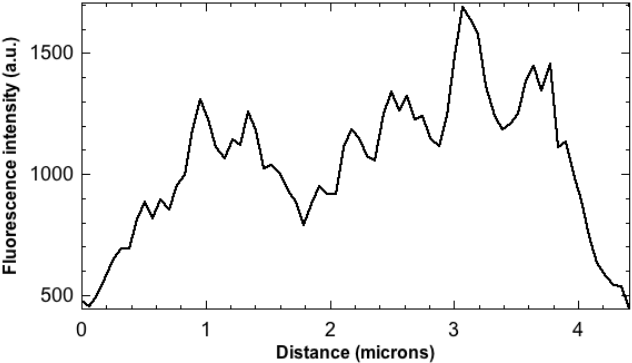

Stack 196

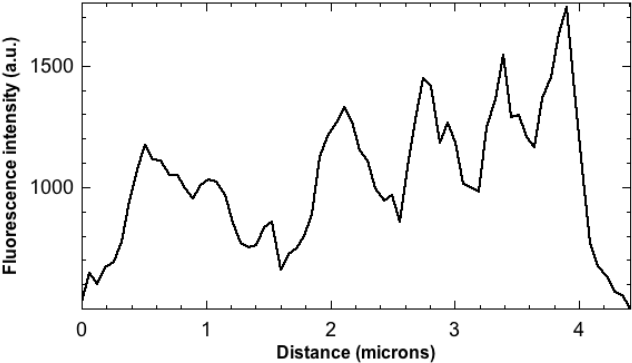

Supplement: FIG S3 [file mBio.03337-19-sf003.pdf]

Supplementary Figure 4

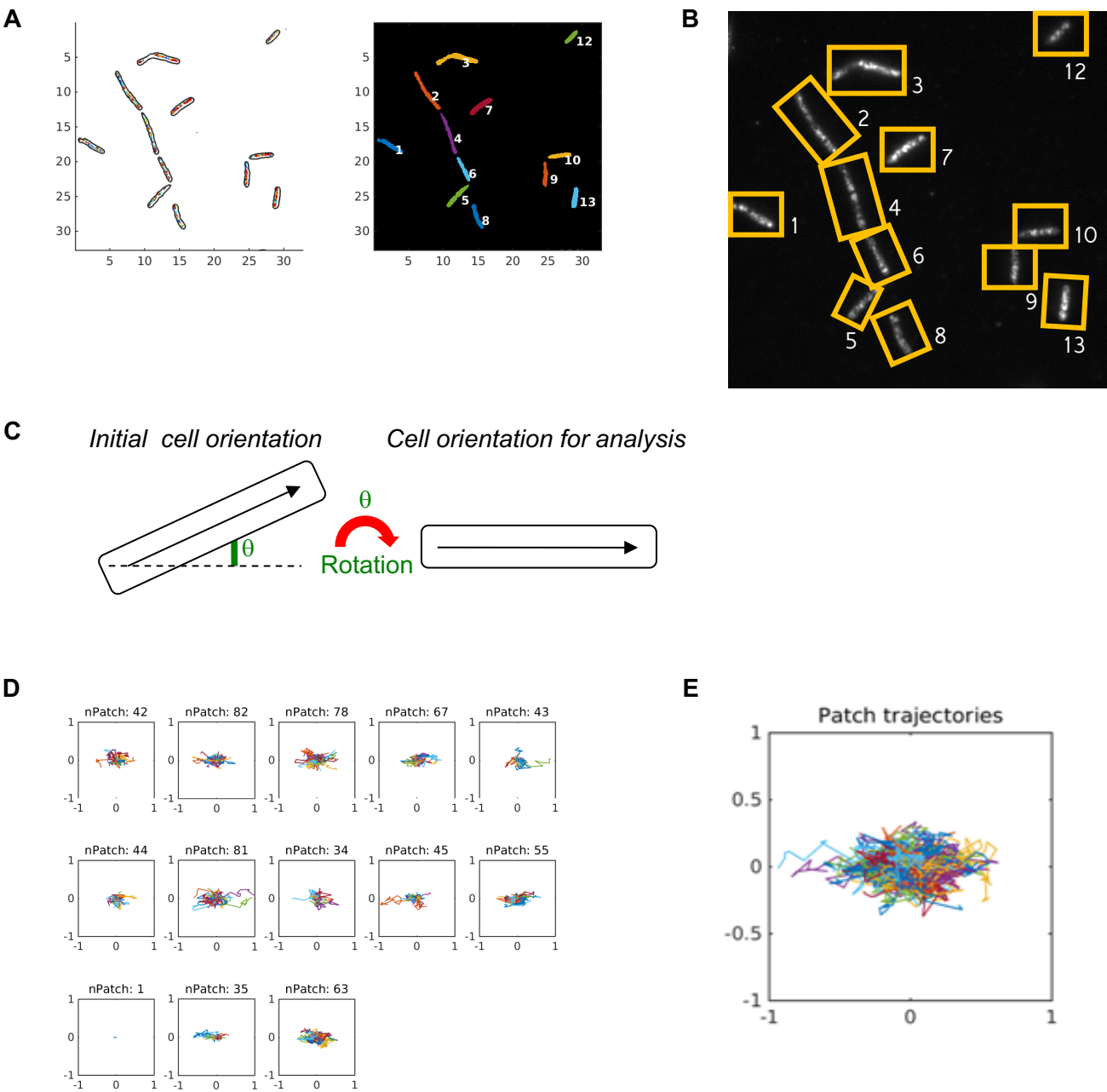

Supplement: FIG S4 [file mBio.03337-19-sf004.pdf]

Supplementary Figure 5

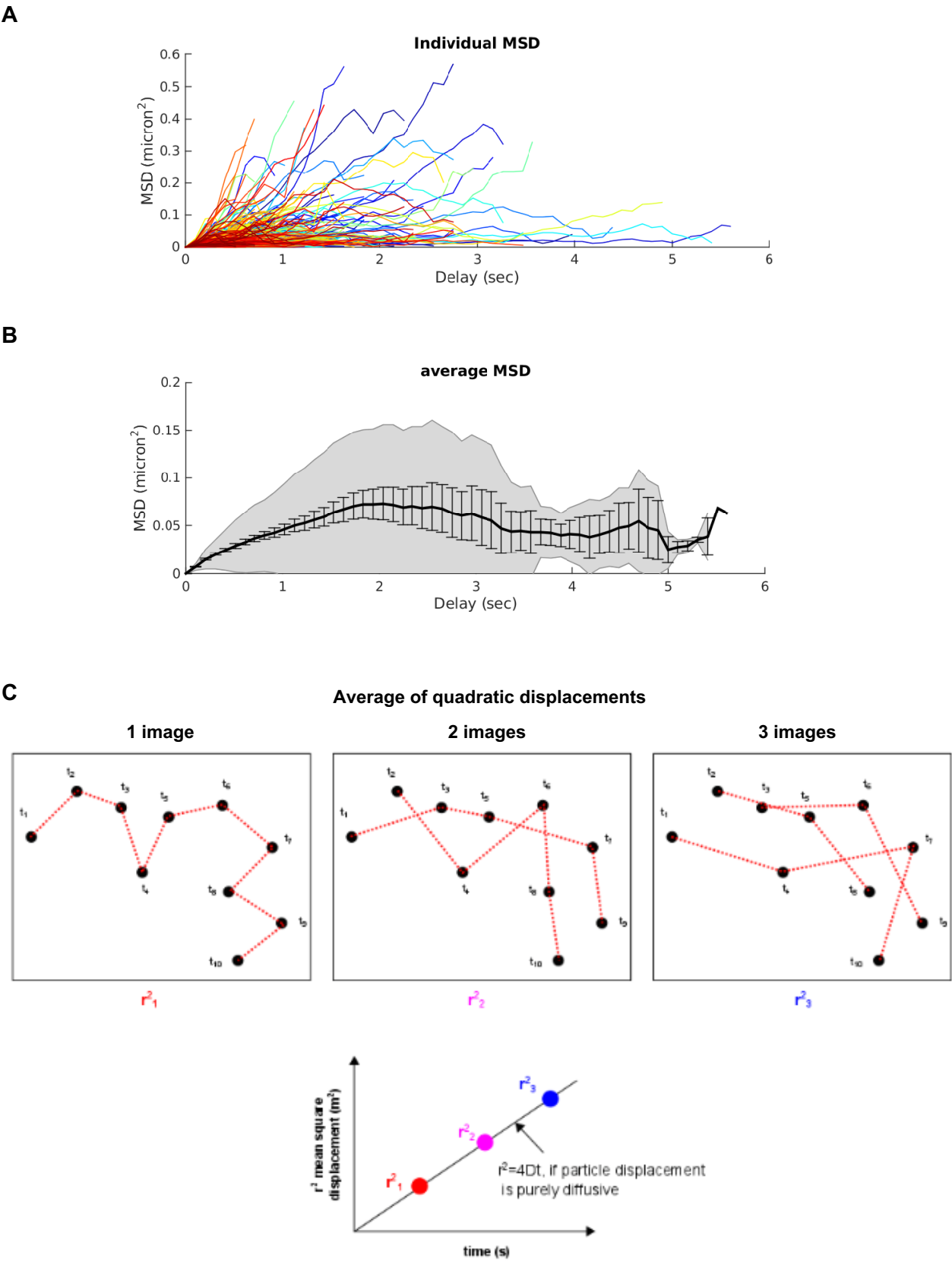

Supplement: FIG S5 [file mBio.03337-19-sf005.pdf]

Supplementary Figure 6

A

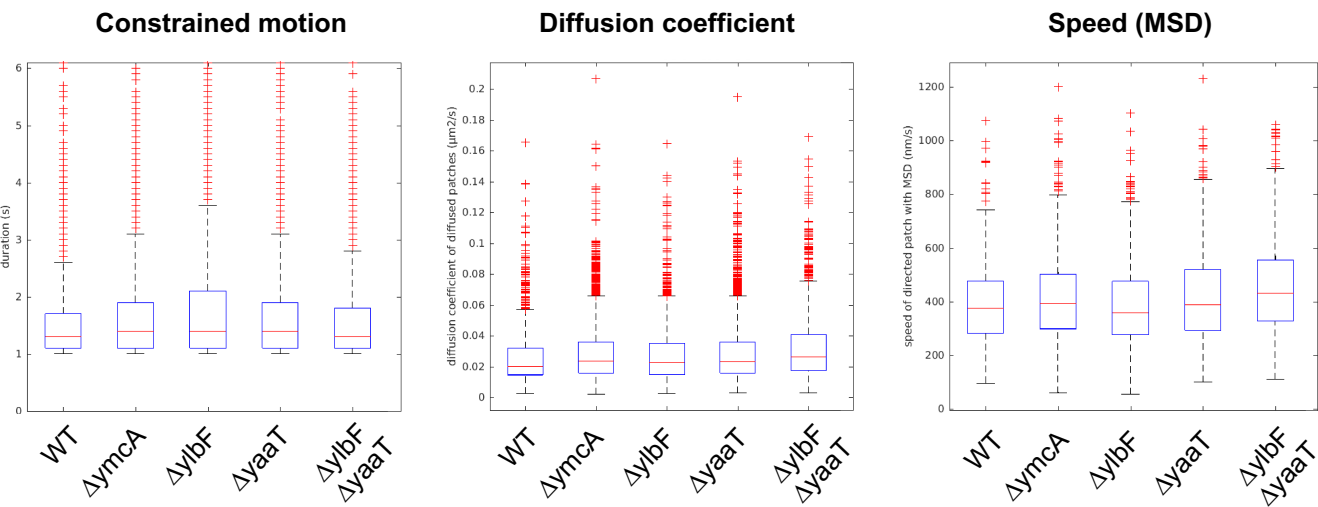

B

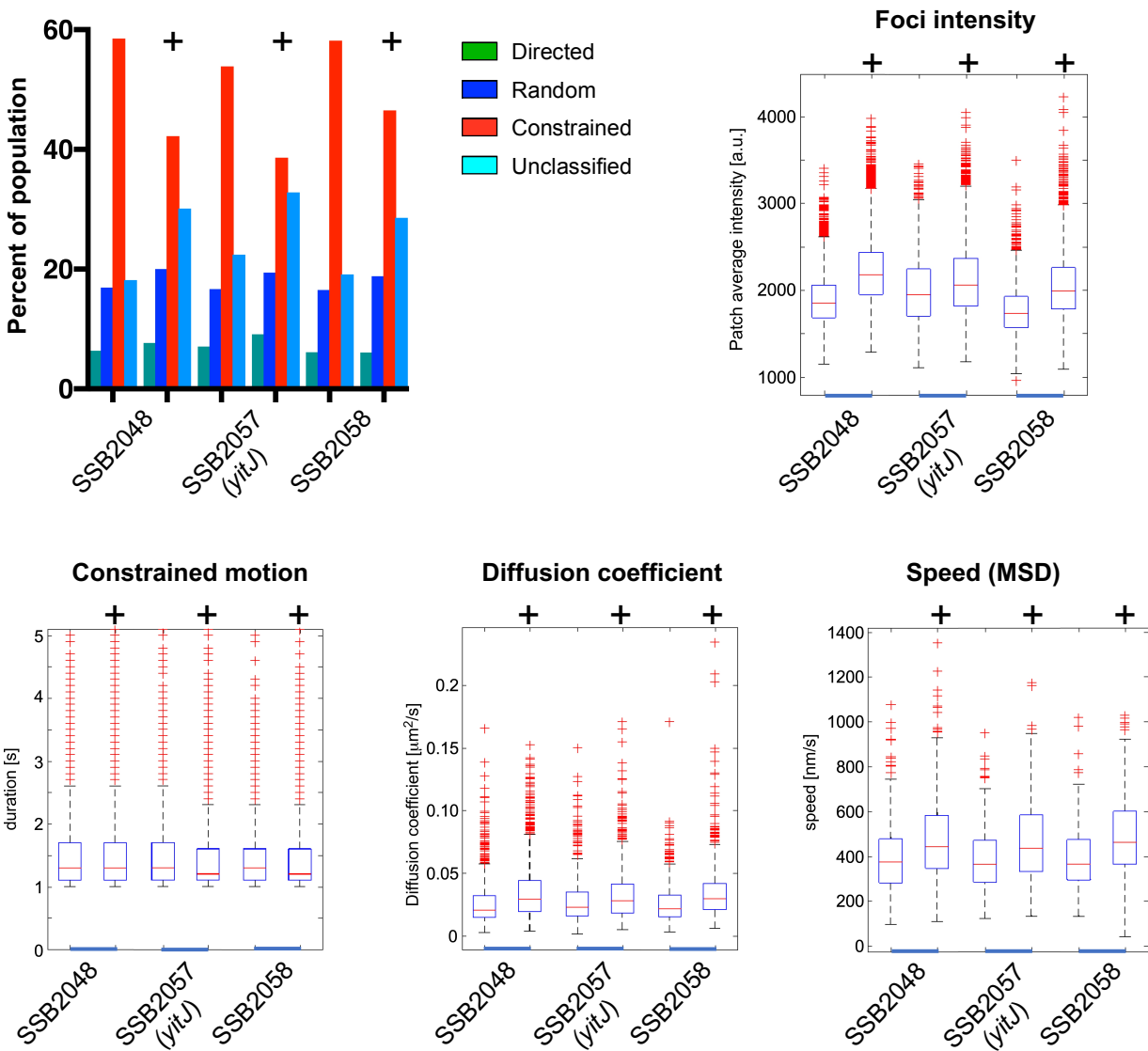

Supplement: FIG S6 [file mBio.03337-19-sf006.pdf]
